# WOMAC and SF-36: instruments for evaluating the health-related quality of life of elderly people with total hip arthroplasty. A descriptive study: WOMAC e SF-36: instrumentos para avaliar a qualidade de vida relacionada à saúde de idosos com artroplastia total de quadril. Um estudo descritivo

**DOI:** 10.1590/1516-3180.2014.8381508

**Published:** 2015-08-03

**Authors:** Mariana Kátia Rampazo-Lacativa, Ariene Angelini dos Santos, Arlete Maria Valente Coimbra, Maria José D’Elboux

**Affiliations:** I MSc. Physiotherapist and Doctoral Student, School of Nursing, Universidade Estadual de Campinas (Unicamp) Campinas, São Paulo, Brazil.; II PhD. Nurse, School of Nursing, Universidade Estadual de Campinas (Unicamp), Campinas, São Paulo, Brazil.; III PhD. Professor, Family Health Program, School of Medical Sciences, Universidade Estadual de Campinas (Unicamp), Campinas, São Paulo, Brazil.; IV PhD. Professor, Postgraduate Gerontology Program, Universidade Estadual de Campinas (Unicamp), Campinas, São Paulo, Brazil.

**Keywords:** Arthroplasty, replacement, hip, Osteoarthritis, hip, Aged, Quality of life, Health of the elderly, Artroplastia de quadril, Osteoartrite do quadril, Idoso, Qualidade de vida, Saúde do idoso

## Abstract

**CONTEXT AND OBJECTIVES:**

Quality-of-life results have increasingly been evaluated among patients undergoing joint replacements. The objective of this study was to compare two assessment instruments for health-related quality of life (one generic and the other specific), among elderly patients undergoing total hip arthroplasty.

**DESIGN AND SETTING:**

Cross-sectional descriptive study in a reference hospital in the region of Campinas.

**METHODS:**

The subjects were 88 elderly outpatients aged 60 years or over who underwent primary total hip arthroplasty. Two instruments for assessing health-related quality of life were applied: the generic Medical Study 36-item Short-Form Health Survey (SF-36) and the specific Western Ontario and McMaster Universities Osteoarthritis Index (WOMAC). Cronbach’s alpha and the ceiling and floor effects of the instruments were evaluated*.*

**RESULTS:**

The scores from both instruments showed that issues of a physical nature affected these elderly people’s quality of life most. The pain and stiffness dimensions of WOMAC showed ceiling effects and only the functional capacity and pain dimensions of the SF-36 did not show the ceiling effect. The SF-36 presented floor effects in the dimensions of physical and emotional aspects. Cronbach’s alpha was considered satisfactory in both instruments (α > 0.70).

**CONCLUSIONS:**

The floor and ceiling effects that were observed suggest that these instruments may present some limitations in detecting changes to the majority of the SF-36 dimensions, except for functional capacity and pain, and to the pain and stiffness dimensions of WOMAC, when applied to elderly people with total hip arthroplasty.

## RESUMO

**CONTEXTO E OBJETIVO:**

Os resultados sobre a qualidade de vida têm sido cada vez mais avaliados em pacientes submetidos a substituições articulares. Este estudo objetivou comparar dois instrumentos de avaliação de qualidade de vida relacionada à saúde (um genérico e outro específico), em pacientes idosos submetidos a artroplastia total de quadril.

**TIPO DE ESTUDO E LOCAL:**

Estudo descritivo transversal em hospital de referência da região de Campinas.

**MÉTODOS:**

Os sujeitos foram 88 pacientes idosos ambulatoriais, com 60 anos ou mais, submetidos a artroplastia total de quadril primária. Foram aplicados dois instrumentos de avaliação de qualidade de vida relacionada à saúde: Medical Study 36-item Short-Form Health Survey (SF-36) (genérico) e Western Ontario and McMaster Universities Osteoarthritis Index (WOMAC) (específico). O coeficiente alfa de Cronbach e os efeitos teto e chão dos instrumentos foram avaliados.

**RESULTADOS:**

Os escores de ambos os instrumentos mostraram que as questões de natureza física são as que mais afetam a qualidade de vida desses idosos. As dimensões dor e rigidez do WOMAC apresentaram efeito teto e apenas as dimensões capacidade funcional e dor do SF- 36 não mostraram o efeito teto. O SF-36 apresentou efeito de chão nas dimensões: aspectos físicos e aspectos emocionais. O coeficiente alfa de Cronbach foi considerado satisfatório nos dois instrumentos (α > 0.70).

**CONCLUSÕES:**

A constatação dos efeitos chão e teto sugere que esses instrumentos podem apresentar algumas limitações, quando aplicados em idosos com artroplastia total de quadril, para detectar alterações na maioria das dimensões do SF-36, exceto capacidade funcional e dor, e nas dimensões dor e rigidez do WOMAC.

## INTRODUCTION

With increasing aging of the population, there is ever-greater prevalence of non-transmissible chronic diseases. Among these, osteoarthritis stands out as one of the most common joint diseases in the elderly population, affecting more than one third of people over 60 years of age.^1^

In Brazil, it is the most common rheumatic disease, responsible for 7.5% of all absences from work, the second most frequent condition in relation to obtaining sickness benefits, and the fourth in determining retirement. Hip osteoarthritis has become a growing problem in Western societies and is a major cause of morbidity and disability among the elderly.^2^

Among the elderly population, there is high incidence of fractures of the proximal femur. It has been estimated that the incidence of this type of fracture will reach 6.3 million cases by 2050, with the growth of older age groups within the world population.^3^ Most hip fractures are caused by falls, and they are a major cause of morbidity and mortality among the elderly. Such fractures are responsible for most of the surgical procedures, including hip replacement, and for most bed occupancy in orthopedic wards.^4^^,^^5^^,^^6^

In the light of these hip disorders affecting the elderly and given the technological advance that have been achieved, total hip arthroplasty has become a widely used method for surgical treatment. This consists of replacing the hip joint and has the purposes of restoring function and promoting pain relief in this joint.^2^^,^^7^

Total hip arthroplasty is called primary when referring to the first surgery on the hip that will be replaced. It has become one of the most common and most successful orthopedic surgical procedures performed today, providing great benefits, especially for the elderly population.^8^^,^^9^

In relation to outcomes used in analyzing the effectiveness of medical treatments or orthopedic surgery, a change has been seen over the last few years. Although clinical changes evaluated through physical and complementary examinations are used in such analyses, health-related quality of life, function, pain scales and satisfaction scales have been emphasized as some of the outcomes from medical and surgical interventions, over recent years. These parameters enable analysis on health status and disease manifestations within an individual’s life from his own subjective perspective, thereby complementing the objective clinical data.^10^

The improvement in health-related quality of life after primary total hip arthroplasty has been documented in several studies.^11^^,^^12^^,^^13^^,^^14^^,^^15^^,^^16^^,^^17^ Concern regarding this issue among the elderly population undergoing this surgical procedure has been highlighted over recent years,^6^^,^^7^^,^^18^^,^^19^^,^^20^ and this shows the importance of such evaluations and clinical research.

With regard to health-related quality of life, two groups of tools stand out: generic tools, which are intended for measuring broader dimensions of quality of life; and specific tools, which are designed for measuring the dimensions of quality of life in specific groups, such as individuals with deformities and disabilities. Both types have advantages and limitations. Among the tools used for evaluating the health-related quality of life in the population with total hip arthroplasty, the generic Medical Study 36-item Short-Form Health Survey (SF-36)^21^ and the specific Western Ontario and McMaster Universities Osteoarthritis Index (WOMAC)^22^ stand out because of their high levels of use.

In the worldwide literature, studies designed to evaluate the psychometric properties of instruments for measuring health-related quality of life among patients with total hip arthroplasty are still at an incipient stage.^23^^,^^24^^,^^25^

With regard to WOMAC, in Brazil, it can be seen that there is a need for this tool to be used more widely in studies evaluating health-related quality of life among patients who undergo total hip arthroplasty, given that in comparison with SF-36, WOMAC has higher sensitivity to small changes in health status, when applied to populations that underwent this surgery.^25^ Furthermore, we found only two studies that assessed the quality of life and function of patients with primary total hip arthroplasty.^26^^,^^27^

Use of a generic tool, complemented by application of a specific tool to assess health-related quality of life when analyzing this type of condition, has the advantage of evaluating the patients’ perception of their general health status combined with the possibility of detecting specific clinical changes to health-related quality of life after primary total hip arthroplasty.^8^^,^^10^^,^^28^

The performance characteristics of generic and specific tools used in the elderly and general populations differ from each other.^29^ Concerning the SF-36, it is important to mention that one study found that this tool is a reliable way of assessing health-related quality of life among elderly patients undergoing primary total hip arthroplasty.^8^ Both SF-36^30^ and WOMAC^31^ have been culturally adapted and validated for the Brazilian context.

## OBJECTIVE

The objective of this study was to evaluate the performance of the Brazilian versions of the SF-36 and WOMAC tools in the elderly population, with regard to ceiling and floor effects and to their internal consistency, as assessed using Cronbach’s alpha.

## METHODS

The subjects of this study were elderly patients of both sexes, aged over 60 years, who underwent primary unilateral total hip arthroplasty and were outpatients at two major referral hospitals in the state of São Paulo, Brazil. The study was approved by the Ethics Committee of each institution and all participants signed a consent form.

Patients were enrolled in the study on the day of their routine outpatient medical visit that had previously been scheduled, to determine the preferences of patients and their companions or caregivers about the best time to make the assessment for the study, i.e. before or after the consultation. Through this, a convenience sample was characterized. We included patients who had undergone unilateral primary total hip arthroplasty at least six months earlier and who were able to understand instructions and to communication verbally. Patients with visual deficits, hemiparesis or hemiplegia, or who had a history of other arthroplasty procedures in another joint that would distort the functioning of the joint now operated, were excluded in order to eliminate the influence of surgical interventions other than total hip arthroplasty on the perception of health-related quality of life. Patients who refused to participate in the study were also excluded.

Data-gathering was performed by the first author of the study, from September 2007 to March 2008. This consisted of consulting the medical records in order to obtain data referring to the patient’s clinical condition and conducting individual interviews to obtain sociodemographic characterizations and measure the health-related quality of life.

### Measurement tools

A sociodemographic and clinical characterization instrument was used to record information on the interviewees relating to social characteristics, health and total hip arthroplasty. This instrument was built specifically for this study and it passed through examination by a professional expert group (physiotherapist, nurse, rheumatologist and orthopedic surgeon). It sought the following sociodemographic data: sex, age, marital status and living arrangements; and information about interviewees’ clinical characteristics: comorbidities, use of medicine, pain in the operated hip, other joint pain, use of walking aids and body mass index. The total hip arthroplasty data included: reason for surgery, hip function evaluated by means of the Harris Hip Score, duration of clinical follow-up, length of postoperative period, type of prosthesis fixation and satisfaction with the results from the surgery.

The Harris Hip Score tool^32^ is an internationally validated hip function evaluation instrument for patients with total hip arthroplasty.^23^ Inclusion of this tool was justified because of its frequent use among local healthcare professionals for functional evaluations of the hip. It consists of a scale on which the total score ranges from 0 to 100 points and the dimensions include pain, function, deformity and range of motion. The maximum score for the pain dimension is 44 points and for the function dimension, 47 points. The latter is subdivided into activities of daily living (ADLs) and walking, with 14 and 33 points respectively. For the deformity dimension, up to four points can be assigned and for the range of motion, up to five points. The functional outcome is considered poor if the total Harris Hip score is less than 70 points. Fair scores are between 70 and 79, good scores are between 80 and 89 and excellent scores are between 90 and 100 points.

The Medical Outcomes Study 36-item Short-Form Health Survey (SF-36)^21^ is a generic tool for assessing health-related quality of life that has been translated and validated for use in Brazil.^30^ It consists of 36 items divided into eight dimensions: functional capacity (ten items), physical aspects (four items), pain (two items), general health status (five items), vitality (four items), social aspects (two items), emotional aspects (three items) and mental health (five items), and one question making a comparative evaluation between current health conditions and the conditions one year ago. The results are evaluated by assigning scores for each question and then transforming the scores into a scale from 0 to 100, on which zero corresponds to “worst health status” and 100 to “best health status”. There are no cutoff points and each dimension is evaluated separately.

The Western Ontario and McMaster Universities Osteoarthritis Index (WOMAC)^22^ is a specific quality-of-life tool for patients with osteoarthritis of hip and knee that has been translated and adapted for use in Brazil.^31^ It is indicated for use in postoperative evaluations on total knee and hip arthroplasty procedures.^23^^,^^25^ This questionnaire was originally developed to be self-administered, but it has been used in interviews and telephone surveys, and, most recently, a computerized version (via e-mail) has also been validated. It comprises 24 items, divided into three dimensions. The pain dimension has five questions, the joint stiffness dimension has two questions and the physical disability dimension has 17 questions. Each question has five possible answers, on a Likert scale (“not at all”, “slightly”, “somewhat”, “moderate” and “extremely”), which are graded 0, 1, 2, 3 and 4, respectively. Thus, zero represents the absence of the symptom and 4 the worst result regarding to that symptom. Through summing the scores, each dimension receives a score that is transformed into a scale of 0 to 100 points, with zero representing the best health status and 100 the worst possible status.

### Statistical analysis

The data were analyzed using SAS for Windows (Statistical Analysis System), version 8.02 (SAS Institute, Inc., Inc., Cary, NC, USA).

Descriptive statistics were used to present the frequency distribution and to calculate the mean, standard deviation, median and range of the sociodemographic and clinical variables and of the data from the health-related quality of life evaluation. To calculate the ceiling and floor effects of the instruments (SF-36 and WOMAC), the proportion of 10% of the best possible score for the tool dimensions was used for the ceiling effect and the proportion of 10% of the worst possible score for the tool dimensions was used for the floor effect.^33^ Presence of a ceiling effect was confirmed when there was asymmetrical distribution of scores and a significant percentage of the population in the study scored at the highest levels for the parameter. Thus, for individuals whose scores were at the extremity of the range of improvement regarding perceptions of health-related quality of life (HRQoL), the instrument would not be able to detect this. The floor effect, in turn, reflected the percentage of subjects whose scores were at the lowest level of the parameter. This type of asymmetrical distribution hampers detection of worsening of perceived HRQoL among the subjects evaluated. This measurement property relates to the ability of the instrument to detect and estimate the magnitude of changes in health condition over time.^33^^,^^34^

The internal consistency was assessed by means of Cronbach’s alpha and α = 0.70 was considered to be satisfactory.^35^

The significance level for statistical tests was 5%, i.e. P < 0.05.

## RESULTS

### Sociodemographic and clinical characterization

Over the study period, 88 patients were enrolled. Among the patients studied, 54.6% were female, most were married and the mean age was 68.8 ± 7.4 years. The average number of comorbidities among the subjects was 3.3 ± 1.5. Forty-nine patients (55.6%) reported pain in the operated hip, but 82 (93.2%) reported pain in other joints. With regard to nutritional status, 23 patients (26.1%) were obese and 49 (55.7%) did not use any walking aid.

The average Harris Hip score was 73.4 ± 19.0, which was considered to be a functional result. However, a greater proportion of elderly patients (39.8%) scored fewer than 70 points, or presented poor hip function (39.8%). It was noteworthy that the most common reason for undergoing surgery among the sample was hip osteoarthritis (84.19%), and these patients remained under clinical follow-up for a mean time of 69.3 ± 55.7 months. The type of fixation used for the prosthesis was most frequently cemented (58.0%) and the mean postoperative period was 59.6 ± 52.4 months (Table 1).

**Table 1.**
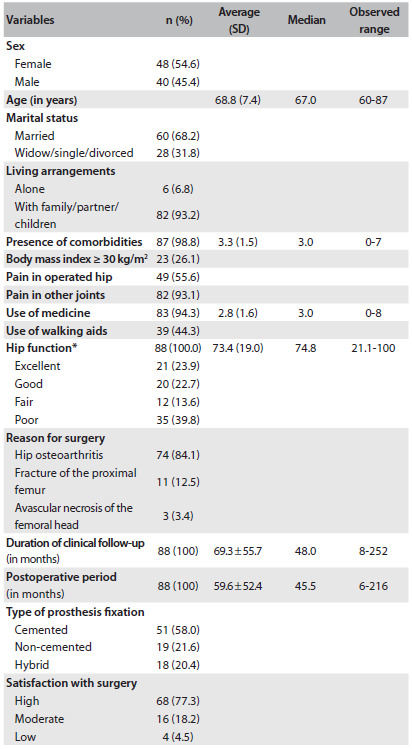
Sociodemographic and clinical characteristics of the total hip arthroplasty subjects studied (n = 88)

SD = standard deviation; *Harris Hip Score.

### Health-related quality of life

Table 2 shows the scores for each dimension of the assessment of health-related quality of life using the SF-36 and WOMAC. Regarding the SF-36, the patients showed higher scores in the dimensions that assess social aspects, vitality and general health status and lower averages in the dimensions that assess physical aspects, functional capacity and pain.

**Table 2.**
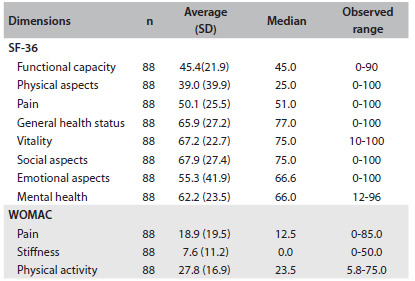
Scores for the dimensions of the SF-36 and WOMAC, for 88 elderly patients with total hip arthroplasty

SF-36 = short-form health survey; SD = standard deviation; WOMAC = Western Ontario and McMaster Universities Osteoarthritis Index; possible variation for each category: 0-100.

In relation to WOMAC, the patients had lower average scores in the dimensions of pain and stiffness, which indicates that pain and stiffness had a minor impact on the quality of life of these elderly people. Although the dimension relating to physical activity had a higher average than the other dimensions, it could be seen from analyzing the observed variation that this dimension did not reach higher scores than those achieved in the pain dimension.

Table 3 describes the mean SF-36 and WOMAC scores of this study and other studies that used these tools. In analyzing the ceiling and floor effects, through measuring the proportion of 10% of the best and worst possible scores obtained on the scales,^35^ these two tools showed a ceiling effect in their dimensions.

**Table 3.**
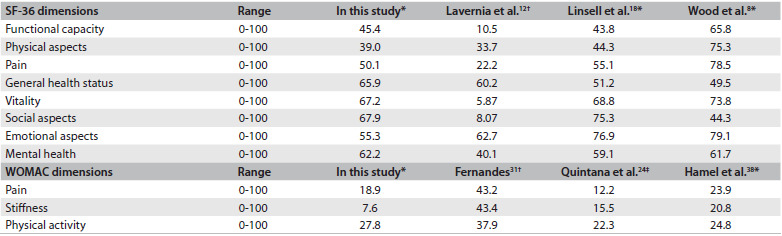
Average scores for SF-36 and Western Ontario and McMaster Universities Osteoarthritis Index (WOMAC) obtained in this study and in other studies

SF-36 = short-form health survey; *Elderly individuals with total hip arthroplasty; ^†^Adults and elderly people with hip osteoarthritis; ^‡^Adults and elderly people with total hip arthroplasty.

WOMAC showed a ceiling effect in the pain and stiffness dimensions, such that the largest population of the patients was concentrated within the stiffness dimension. Only the functional capacity and pain dimensions of the SF-36 did not show the ceiling effect. Among the other dimensions, which presented the ceiling effect, higher proportions of patients were found in the dimensions of emotional, social and physical aspects and vitality. The SF-36 also presented floor effects regarding physical and emotional aspects (Table 4).

**Table 4.**
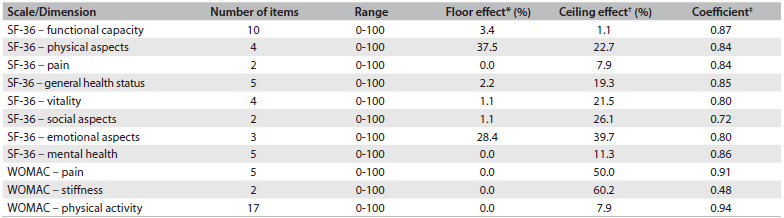
Descriptive analysis of the floor and ceiling effects obtained in this study

SF-36 = short-form health survey; WOMAC = Western Ontario and McMaster Universities Osteoarthritis Index; *Floor effect equivalent to 10% of the worst possible results of the scale; ^†^Ceiling effect equivalent to 10% of the best possible results of the scale;^33^^‡^Cronbach’s alpha coefficient for Likert scales.

In comparing the ceiling and floor effects of these two tools with the hip function of the Harris Hip Score, patients who scored within the ceiling effect, in both the WOMAC and the SF-36 tools, showed better hip function (P < 0.05). On the other hand, those who scored within the floor effect of the SF-36 showed worse hip function (P = 0.01).

The reliability of the WOMAC and the SF-36 tools, as evaluated according to their internal consistency, was satisfactory. All the dimensions presented values greater than 0.7, except for the WOMAC stiffness dimension (Table 4).

## DISCUSSION

Regarding the SF-36, higher scores that indicated better quality of life were observed in the dimensions of social aspects, vitality, general health and mental health status, while lower scores were observed in the dimensions of physical aspects and functional capacity.

In applying WOMAC in relation to the physical dimension, those who referred to physical activities of daily life showed a higher mean score for this than for the other dimensions, thus indicating that these patients’ quality of life seemed to be more related to the difficulty of performing everyday activities. These data were similar to those of a study in which the subjects also had higher scores in the physical activity dimension.^24^^,^^36^ Other studies have described similar results.^12^^,^^13^^,^^37^^,^^38^

Previous studies have described health-related quality of life among elderly people with total hip arthroplasty.^8^^,^^18^ While there are some results that resemble those of the present study, i.e. lower scores in the functional capacity and physical appearance dimensions,^18^ the authors Wood and McLauchlan found lower means for the general health and mental health status dimensions of the SF-36.^8^ Studies on elderly populations with other chronic diseases have described functional ability and physical aspects as the dimensions of the SF-36 that most affect health-related quality of life.^39^^,^^40^ Thus, the findings from the present survey show that, although this population had undergone a surgical intervention with the aim of functional improvement, physical problems were still the ones that most affected health-related quality of life among elderly patients with total hip arthroplasty.

In this study, WOMAC showed a ceiling effect in the pain and stiffness dimensions, with a greater proportion than what was shown in another investigation that also found a ceiling effect in the physical activity dimension.^16^ Another similarity was the absence of floor effects in all the dimensions of WOMAC.

In contrast, in a study on a sample of 469 subjects with total hip arthroplasty, WOMAC presented floor effects in the stiffness and pain dimensions and did not show ceiling effects in any of the three dimensions.^24^ The presence of a ceiling effect in the pain and stiffness dimensions may indicate that these dimensions are probably not capturing changes in the population studied, which suggests that the tool may be “unable to measure” or “measures very little of” patients’ improvements in these dimensions.

With regard to the SF-36, floor effects were found in the physical and emotional aspect dimensions. These data were similar to those of another study,^24^ but with smaller proportions of subjects. Ceiling effects occurred in the physical, social and emotional aspect dimensions in the earlier study after total hip arthroplasty, thus confirming the data from the present study, which in addition to these dimensions, also showed ceiling effects in relation to the general health status and vitality dimensions. In another study, no effect was detected through the SF-12, which thus indicates that this tool had better performance with regard to picking up positive or negative changes in patients with total hip arthroplasty.^16^

According to some authors,^34^ the existence of ceiling and floor effects in the tools indicates that their items or scales may have difficulty in discriminating between the subjects and, therefore, present reduced sensitivity and responsiveness. Consequently, patients whose scores are within the floor effect of a tool may be in “such a bad” condition that the tool is unable to detect worsening of their condition. On the other hand, patients whose scores are within the ceiling effect of a tool show little possibility of improvement, thus indicating that the tool is unable to detect improvement. In the present investigation, the floor and ceiling effects obtained seemed to indicate that some issues among the population studied, namely elderly patients undergoing total hip arthroplasty, were handled inadequately.

By comparing the floor and ceiling effects of the WOMAC and SF-36 with hip function evaluated using the Harris Hip Score, it was clear that patients with better functional scores showed a ceiling effect, and patients who were considered to be functionally worse had scores within the floor effect. This result shows that patients with minor limitations were concentrated among the best possible scores of the tools, while those with major limitations showed the worst possible scores of the tools.

The internal consistency of the WOMAC and SF-36 tools was satisfactory. The Cronbach’s alpha values were greater than 0.90 for the pain and physical activity dimensions of WOMAC. These data are similar to those of previous studies that showed satisfactory reliability for this tool; however, in the pain dimension, the alpha values were greater than 0.80.^24^^,^^41^

In the stiffness dimension, the Cronbach’s alpha value was 0.48, which does not correspond with findings from other studies,^24^^,^^41^ which presented values greater than 0.80. This divergence may have occurred because the population of the present study was exclusively elderly, which would therefore indicate that this tool showed different behavior for this population. The inclusion criteria may also have contributed, since the sample consisted of subjects who theoretically had resolved their issue of stiffness through the surgery. Moreover, these earlier studies^24^^,^^41^ were not composed solely of elderly patients because age was not an inclusion criterion.

Regarding the SF-36, all its dimensions presented Cronbach’s alpha values greater than 0.70, as also seen in some other studies,^23^^,^^24^^,^^41^ except for the last these, which showed an alpha of 0.67 in the pain dimension, although this was close to the criterion of satisfactory. While investigating the internal consistency of this tool in different groups of elderly people, we found that it had presented satisfactory reliability when used among elderly patients on hemodialysis and among elderly patients with heart failure.^38^^,^^42^

Some limitations were identified in this study. The number of subjects was relatively small. The criteria for inclusion and exclusion adopted reduced the chances of a larger sample, but selected a homogeneous group of subjects in relation to surgery. Another important point was that the descriptive nature of this study did not allow us to say which instruments for assessing health-related quality of life among elderly patients with total hip arthroplasty were the most appropriate. Further investigations with more significant numbers of subjects, longitudinal designs and deeper statistical approaches are needed, in order to achieve better understanding of the performance of the SF-36 and WOMAC instruments when applied to elderly patients after this surgery.

The analysis accomplished in this study was consistent with the recommendations in the literature regarding concomitant use of specific and generic tools for measuring health-related quality of life. Furthermore, the physical, psychological and social particularities of aging should be taken into consideration in clinical practice, so as to choose tools capable of assessing health-related quality of life when investigating elderly populations with total hip arthroplasty.

## CONCLUSION

The WOMAC and SF-36 tools presented satisfactory levels of reliability in this sample, However, only the functional capacity and pain dimensions of the SF-36 showed no ceiling effect, and floor effects were detected in the dimensions of physical and emotional aspects. These findings show that these dimensions have little sensitivity for detecting changes in the group evaluated. This study also confirmed the presence of a ceiling effect relating to the WOMAC pain and stiffness dimensions. The observation of floor and ceiling effects in using these tools suggests that they may present some limitations when applied to elderly patients with total hip arthroplasty.
